# Cost-Effectiveness of Digital Breast Tomosynthesis vs. Abbreviated Breast MRI for Screening Women with Intermediate Risk of Breast Cancer—How Low-Cost Must MRI Be?

**DOI:** 10.3390/cancers13061241

**Published:** 2021-03-12

**Authors:** Fabian Tollens, Pascal A.T. Baltzer, Matthias Dietzel, Johannes Rübenthaler, Matthias F. Froelich, Clemens G. Kaiser

**Affiliations:** 1 Department of Radiology and Nuclear Medicine, University Medical Centre Mannheim, Medical Faculty Mannheim, University of Heidelberg, Theodor-Kutzer-Ufer 1-3, 68167 Mannheim, Germany; fabian.tollens@medma.uni-heidelberg.de (F.T.); matthias.froelich@medma.uni-heidelberg.de (M.F.F.); 2 Department of Biomedical Imaging and Image-Guided Therapy, Vienna General Hospital, Medical University of Vienna, 1090 Wien, Austria; pascal.baltzer@meduniwien.ac.at; 3 Department of Radiology, Friedrich-Alexander-University Hospital Erlangen, 91054 Erlangen, Germany; matthias.dietzel@uk-erlangen.de; 4 Department of Radiology, Ludwig-Maximilians-University Munich, 80331 München, Germany; rjohannes.ruebenthaler@med.uni-muenchen.de

**Keywords:** tomosynthesis, AB-MRI, MR-Mammography, breast cancer, intermediate-risk screening, cost-effectiveness analyses, cost-effectiveness threshold

## Abstract

**Simple Summary:**

Women with dense breasts have an increased risk of breast cancer and a smaller likelihood of detection in conventional mammographic screening. It is an ongoing challenge of breast imaging techniques to increase sensitivity in dense breasts. Tomosynthesis, as well as abbreviated breast MR, intends to close the gap between optimal cancer detection rate and cost-effectiveness. The aim of this economic evaluation was to analyse the cost-effectiveness of these two imaging techniques in screening women of intermediate risk of breast cancer due to dense breasts. The model-based analysis revealed that abbreviated breast MRI can be considered cost-effective, being below the willingness to pay-threshold of $100,000 per quality-adjusted life-year gained.

**Abstract:**

Background: Digital breast tomosynthesis (DBT) and abbreviated breast MRI (AB-MRI) offer superior diagnostic performance compared to conventional mammography in screening women with intermediate risk of breast cancer due to dense breast tissue. The aim of this model-based economic evaluation was to analyze whether AB-MRI is cost-effective in this cohort compared to DBT. Methods: Decision analysis and Markov simulations were used to model the cumulative costs and quality-adjusted life-years (QALYs) over a time horizon of 30 years. Model input parameters were adopted from recent literature. Deterministic and probabilistic sensitivity analyses were applied to test the stability of the model. Results: In the base-case scenario, the costs of an AB-MRI examination were defined to equal the costs of a full protocol acquisition. Two-yearly screening of women with dense breasts resulted in cumulative discounted costs of $8798 and $9505 for DBT and AB-MRI, and cumulative discounted effects of 19.23 and 19.27 QALYs, respectively, with an incremental cost-effectiveness ratio of $20,807 per QALY gained in the base-case scenario. By reducing the cost of an AB-MRI examination below a threshold of $241 in sensitivity analyses, AB-MRI would become cost-saving compared to DBT. Conclusion: In comparison to DBT, AB-MRI can be considered cost-effective up to a price per examination of $593 in screening patients at intermediate risk of breast cancer.

## 1. Introduction

Up to this day, mammography is the main diagnostic pillar of breast cancer screening in women with average risk of breast cancer worldwide. However, considering the age groups eligible for screening, almost half of the women have heterogeneously or extremely dense breast tissue [1].

Dense breasts are associated with an increased risk of breast cancer and a reduced likelihood of breast cancer detection on conventional mammography [2,3,4]. In order to improve the diagnostic performance of x-ray-based methods, digital breast tomosynthesis (DBT) is increasingly being applied either in addition to or as a replacement for breast cancer screening. The 3-dimensional mammographic imaging in DBT offers increased cancer detection rates and reduced false-positive findings [5,6].

MR-mammography (MRM) is the most sensitive technique in breast cancer detection. Diagnostic performances with sensitivities above 95% and specificities of about 95% have consistently been reported [7,8]. Recent prospective multicenter studies could not show a substantial diagnostic benefit of conventional imaging, even in combination for high-risk patients [9,10,11], when MRM had additionally been performed.

However, concerns about the cost of MRM, as well as the discussion about its specificity in non-expert hands, have confined it to the screening of high-risk patients or as problem solver in individual cases [12].

The benefits of extending the use of MRM to screening women with intermediate and average risk of breast cancer have been indicated by an increasing number of studies [4,13,14]. At the same time, the cost-effectiveness of MRM in screening women at intermediate risk of breast cancer has recently been shown with an incremental cost-effectiveness ratio (ICER) far below commonly accepted willingness to pay (WTP)-thresholds [15,16].

Nevertheless, the abbreviation of MRM protocols (AB-MRI) with the aim of reducing both acquisition and image reading times has been in the scientific focus in the previous years in order to find the optimal balance between cost and diagnostic performance [17]. Abbreviated protocols are supposed to improve the cost-effectiveness of breast MRI, particularly in populations that are not at high risk of breast cancer and in health care systems with conservative WTP-thresholds.

Recently, the first prospective head-to-head comparison of DBT and AB-MRI for screening patients at intermediate risk of breast cancer has shown the superior diagnostic value of AB-MRI [14]. In light of evolving data in new patient collectives, we intend to perform an economic evaluation of the cost-effectiveness of DBT vs. AB-MRI from a U.S. healthcare system perspective in order to address the following questions:

1. Compared to DBT, is AB-MRI a cost-effective modality in screening women with dense breasts for breast cancer?
2. Considering the cost benefits by abbreviating breast MRI: How cheap do we have to abbreviate MRI in order to compensate for the diagnostic difference?

## 2. Results

### 2.1. Cost-Effectiveness Analysis

Over a time horizon of 30 years, the simulation of long-term costs and effects resulted in average cumulative costs of $8798 and an average cumulative effect of 19.23 quality-adjusted life-years (QALYs) for DBT (Table 1). In contrast, using AB-MRI resulted in average costs of $9505 and a cumulative quality of life of 19.27 QALYs. Even though the average results per woman might naturally appear small on a population level, the individual benefits, i.e., gains in QALYs for affected women, may be substantial. The consecutive ICER of AB-MRI was $20,807 per QALY (Table 1), below the accepted willingness-to-pay-threshold of $100,000 per QALY. Therefore, the use of AB-MRI in screening women with dense breasts for breast cancer may be regarded cost-effective at the selected WTP-threshold in the base-case scenario.

### 2.2. Sensitivity Analysis

#### 2.2.1. Deterministic Sensitivity Analysis

**Figure 1:**
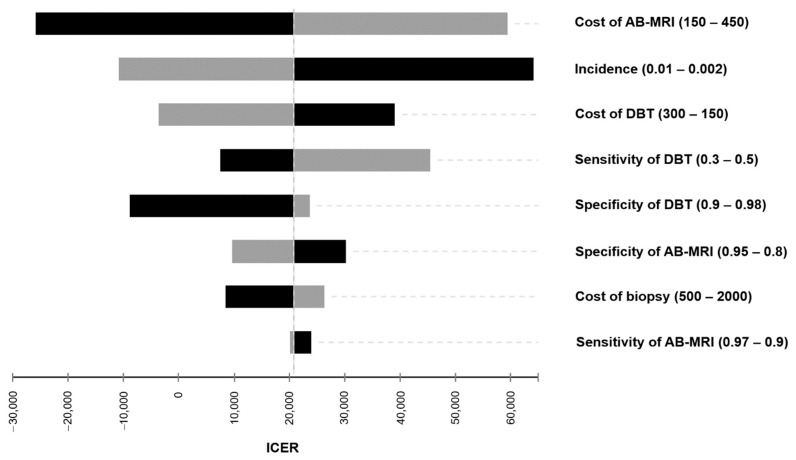
Tornado diagram of the one-way sensitivity analysis. Input parameters were varied within a reasonable range to indicate their impact on the incremental cost-effectiveness ratio (ICER) in US-$ per QALY gained.

The results of the one-way sensitivity analysis are illustrated in Figure 1 and Table S1. Cost of AB-MRI and incidence were identified as the most influential drivers of cost-effectiveness. Considering diagnostic factors with the most impact on the ICER, the sensitivity of DBT was the biggest driver of cost-effectiveness. A variation of all input parameters within reasonable ranges resulted in AB-MRI consistently remaining cost-effective with ICER values below a WTP-threshold of $100,000.

#### 2.2.2. Costs of AB-MRI

**Figure 2:**
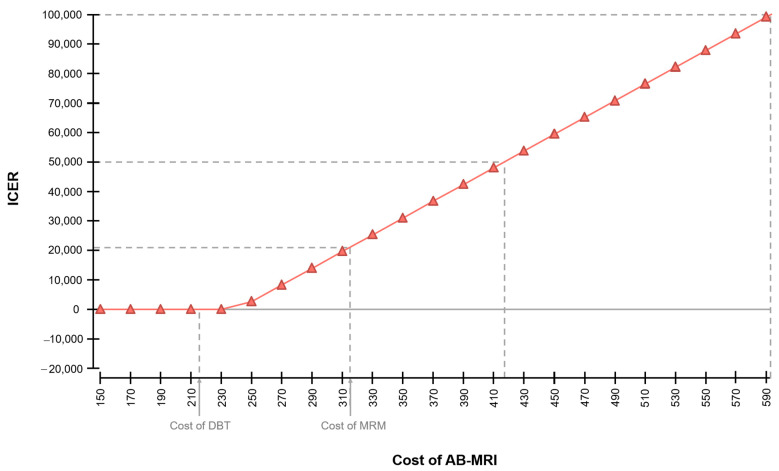
One-way sensitivity analysis for the cost of AB-MRI (US-$) and the corresponding incremental cost-effectiveness ratio (ICER in US-$ per QALY gained) compared to DBT. Screening with AB-MRI becomes cost-saving when the cost per examination of AB-MRI is reduced below $240.79.

In the base case scenario, the costs of AB-MRI were defined to equal the costs of a full protocol breast MRI examination. The average cumulative costs of an AB-MRI screening program over a time horizon of 30 years exceeded the costs of DBT by $706 (Table 1). The cost of an AB-MRI examination was set to $314 compared to $214 for DBT (Table 2). Variations of the costs of MRI were associated with significant changes of the resulting overall cost-effectiveness. Decreasing costs of AB-MRI were associated with a smaller corresponding ICER (Figure 2). Up to examination cost of $593 for AB-MRI, the resulting ICER fell below a hypothetical WTP-threshold of $100,000. Below AB-MRI examination costs of $240.79, screening with AB-MRI may be assumed to not only be diagnostically superior, but also cheaper. The resulting cumulative costs and ICERs for varying costs of AB-MRI and DBT are reported in Table 3.

#### 2.2.3. Probabilistic Sensitivity Analysis

**Figure 3:**
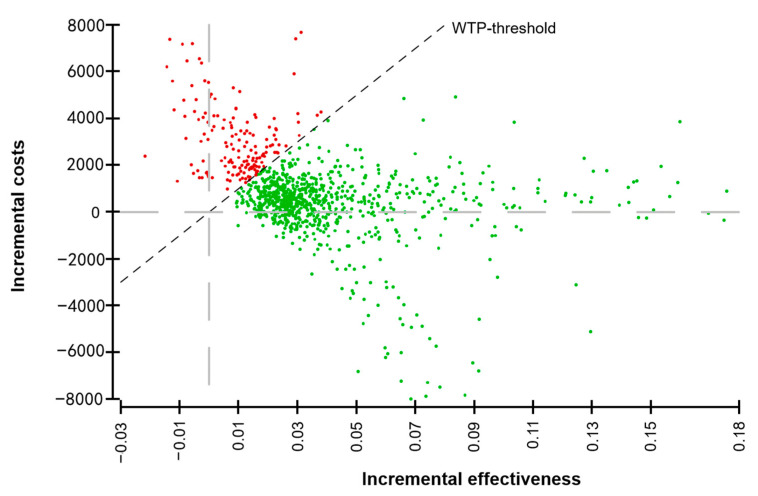
Cost-effectiveness scatter plot. Incremental costs (US-$) and effectiveness (quality-adjusted life-years, QALYs) when comparing AB-MRI to DBT as determined in a Monte Carlo simulation. The majority of the iterations fall below the willingness to pay (WTP)-threshold of $100,000 per QALY.

The distribution of the resulting ICER-values based on the probabilistic sensitivity analysis and Monte Carlo simulation is shown in Figure 3. The majority of iterations resulted in higher average costs of AB-MRM and higher average outcomes.

**Figure 4:**
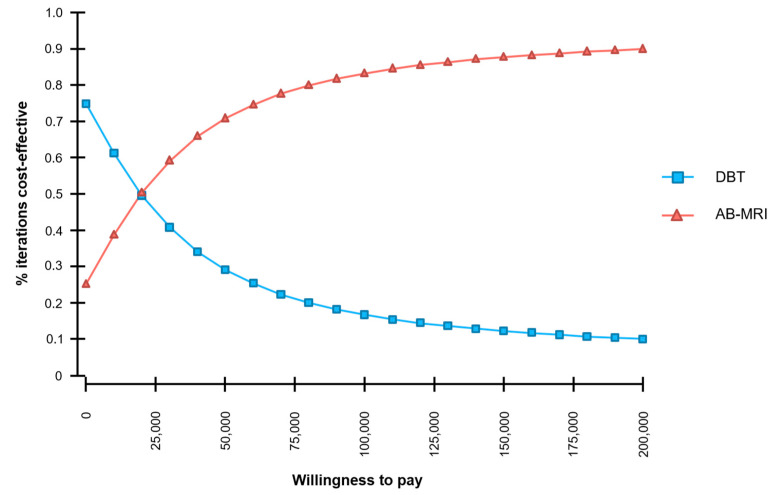
Cost-effectiveness acceptability curve based on a probabilistic sensitivity analysis and Monte Carlo simulation with 30,000 iterations. At a willingness to pay (WTP)-threshold of $100,000 per QALY, AB-MRI was cost-effective in 83.2% of the iterations.

The stability of the model is illustrated by a cost-effectiveness acceptability curve (Figure 4 and Table S2). At a WTP-threshold of $100,000, 83.2% of the simulations were cost-effective.

## 3. Discussion

In 2014, Kuhl et al. first introduced the concept of abbreviated breast MRI as a means to significantly reduce costs of MRM while maintaining an acceptable specificity [17]. In the following years, various protocols have been proposed: Non-contrast T1- and T2-weighted images followed by contrast-enhanced images, ultrafast contrast-enhanced sequences, or diffusion-weighted images as a contrast-free alternative [18,19,20,21,22,23].

At the same time, DBT was introduced in order to enhance the diagnostic performance of conventional mammography [5,24,25] and is now widely implemented in place of two-dimensional mammography.

While literature reveals a better diagnostic performance of AB-MRI [17], DBT may be significantly less expensive per examination, raising the question as to their incremental cost-effectiveness.

Recently, Comstock et al. have published the first head-to-head comparison of DBT and AB-MRI in a screening setting for women of intermediate risk of breast cancer due to their elevated levels of breast density [14]. The study’s multicenter approach addressed abbreviated protocols of various composition and length, yet all including T2-weighted and pre- and post-contrast T1-weighted images and a time limit of 10 min per examination. This data on the comparative diagnostic performance of the two screening methods now unprecedentedly allows for the economic evaluation of cost-effectiveness.

In our model-based evaluation, we are able to demonstrate that AB-MRI is not only the diagnostically superior strategy in terms of long-term outcomes, but also the more cost-effective alternative in screening women in this specific screening cohort—below the WTP-threshold of most westernized countries as described above. Our results, therefore, deem AB-MRI to be the superior screening technique in dense breasts, especially considering MRM to be a radiation-free technique.

However, the drawbacks of abbreviating MRM need to be discussed in detail: Even though Kuhl et al. initially defined the use of abbreviated protocols, current data suggest variations to be accepted forms of abbreviation (i.e., additional T2 weighted images, etc.). Depending on the composition of the abbreviated protocols, variations of the diagnostic performances in terms of specificity and various effects on cost-effectiveness must be assumed.

Further studies are required in order to examine the optimal cut-off between accuracy and costs, also with medical and economic consequences of future diagnostic or histologic clarification of unclear findings in mind (recall rates). Standardization of abbreviated protocols will ensure optimal quality of breast MRI as a screening tool.

Currently, there is no consented cost estimation or Medicare CPT code for AB-MRI. Therefore, in the base-case scenario of our analysis, the costs of AB-MRI were defined to equal the costs of a full protocol.

In the next step, the impact of varying prices per examination of AB-MRI on the overall cost-effectiveness was examined in sensitivity analyses in order to consider a range of possible cost constellations (Figure 2 and Table 3). The cost of AB-MRI was identified as one of the key determinants of cost-effectiveness in the general sensitivity analysis (Figure 1).

By reducing the cost per examination of AB-MRI below $241, the technique becomes cost-saving compared to DBT—due to the superior diagnostic performance of AB-MRI compared to DBT as well as its lower price in this case. Assuming a reasonable range of costs per examination up to $314 (current Medicare average cost of the full protocol MRI-examination), the ICER increases to $20,807 per QALY within this range.

Prior studies have demonstrated that full MRM-protocols can be considered cost-effective compared to conventional mammography in screening women at intermediate risk of breast cancer with an incremental cost-effectiveness ratio of $ 8797.60 per QALY gained [15,16]. The authors believe it may be important to emphasize that “full protocols” are equally undefined in their composition up to this point as AB-MRI protocols, also leaving a certain spectrum for economical interpretation.

Further studies should compare the diagnostic performance of varying AB-MRI- as well as varying full-MRM protocols with their consecutive economic effects, also in further patient cohorts, such as women of average risk and women with less dense breast parenchyma (density categories A & B) as opposed to extremely dense breast tissue (category D) in order to identify the diagnostic benefit for different risk-stratified subgroups.

The model-based economic evaluation is afflicted with certain limitations:

The follow-up interval reported by Comstock et al. was below two years, and long-term data on the diagnostic performance of the imaging modalities in subsequent screening rounds are not available so far.

The data used as input parameters for this study was adopted from published literature. In line with common practice in conducting economic evaluations, the corresponding authors of underlying literature were not contacted individually. Even though common practice, this may be considered a possible weakness of our study.

The United States healthcare system perspective has been selected for this economic evaluation for the purpose of standardization and comparability to other available literature. It would obviously be important to interpret our results in the light of varying input parameters of different healthcare systems, such as Europe or Asia. Screening methods, costs, and QOL might differ in other countries. However, the authors do not believe that the impact of effects to be significantly altered by international differences.

Our underlying Markov model included various possible stages of local and regional breast cancer to reflect the diversity of different stages of the disease upon detection in a screening program. Throughout the time frame of 30 years, false-positive and false-negative findings and their corresponding costs and impairments of QOL were included in the simulations as well as true positives and true negatives. Variations of the incidence of breast cancer resulted in significant effects on cost-effectiveness, which confirms the validity of the Markov model design and the assumptions in our model-based analysis. However, a Markov model can only represent simplified pathways of cancer detection and subsequent treatment. It will never accurately reflect any possible manifestation of clinical reality.

## 4. Materials and Methods

### 4.1. Screening Collective

The economic evaluation was conducted based on published data of a study investigating 1516 women at intermediate risk of breast cancer (median age of 54 years, range 40–75 years) [14], resembling an unprecedented study population. The multicenter program was dedicated to women with dense breasts (ACR BI-RADS categories C or D) and evaluated the diagnostic performance of DBT and AB-MRI. The cross-sectional study with longitudinal follow-up at 11–13 months after the study baseline was conducted at 47 institutions in the United states and 1 institution in Germany.

### 4.2. Cost-Effectiveness Modelling

#### 4.2.1. Decision Model

**Figure 5:**
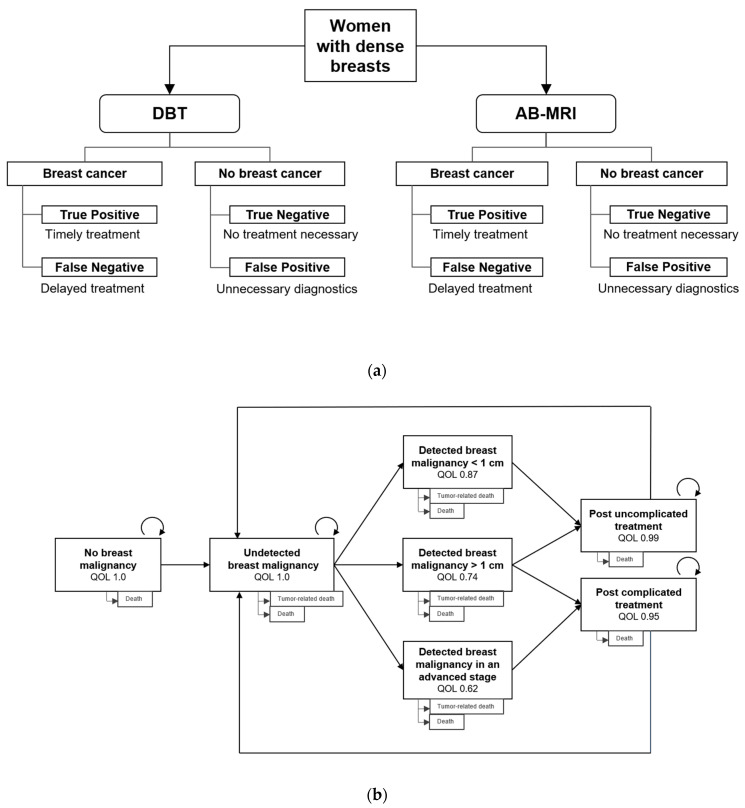
(**a**) Decision-tree including both screening strategies and the corresponding outcomes. (**b**) Markov Model: Probability of breast malignancy detection depends on the sensitivity of the screening modality and the time since development of the disease. Corresponding quality of life (QOL) for the Markov states is indicated in the boxes.

To compare the diagnostic strategies, a decision analytic model including DBT and AB-MRI was constructed, and the respective outcomes true-positive, false-positive, true negative, and false-negative were defined for each diagnostic strategy (Figure 5a). The probability of each diagnostic result depended on the performance of the diagnostic modality as determined in the screening program [14].

#### 4.2.2. Markov Model

To model long-term costs and outcomes of screening women for breast cancer, the Markov model designed by the publishing research group [16] was used and refined to reflect the characteristics of the screening collective and a two-yearly screening program (Figure 5b). Markov states were constructed to represent the absence of cancer, undetected breast cancer, detected malignancy, post-treatment states, and death. The cycle length was set to one year so that each Markov state was characterized by its annual costs and the associated quality of life (QoL). The screening interval was set to 2 years. A true-positive finding resulted in a timely treatment, whereas a false-negative finding consecutively resulted in delayed treatment and a higher probability of progressive disease with more extensive and costly therapy. In case of false-positive findings, unnecessary follow-ups with associated costs and impairment of quality of life (QoL) were assumed. Positive findings of DBT resulted in biopsy, whereas positive findings of AB-MRI resulted in a full protocol breast MRI examination followed by a biopsy only in case of a confirmed finding. Timely detection of breast malignancies depended on the sensitivity of the applied screening modality.

### 4.3. Input Parameters

For this economic evaluation comparing DBT and AB-MRI in women with dense breasts, relevant input parameters were extracted from the literature (Table 2), closely following international recommendations on the conduct of cost-effectiveness analyses [26].

#### 4.3.1. Diagnostic Efficacy Parameters

The diagnostic performance of DBT and AB-MRI in a screening setting for women with dense breasts was recently compared [14]. Sensitivities of 95.7% and 39.1% and specificities of 86.7% and 97.4% were reported for AB-MRI and DBT, respectively.

#### 4.3.2. Utilities

Quality of life was estimated for each Markov state based on available literature. To account for treatment-related differences in the QOL depending on the size of the tumor at the time of detection, a utility of 0.87 was applied for tumors smaller than 1 cm, a utility of 0.74 for tumors larger than 1 cm and a utility of 0.62 for regional breast cancer in an advanced stage [27,28,29]. Extensive and systemic treatment of large tumors was assumed to result in reduced QOL in the long term (0.95) compared to the QOL after treatment of localized disease (0.99). Women without detected breast cancer were assumed to have unimpaired QOL.

#### 4.3.3. Cost Estimates

Costs of the diagnostic procedures were collected based on the Medicare-specific Current Procedural Terminology (CPT) codes of each modality and on reported average Medicare costs [24,30]. For AB-MRI examinations, Medicare costs of an MRI examination of both breasts were applied. Potential savings in MRI examination costs were evaluated in the sensitivity analysis (Figure 2). Treatment costs from a Medicare setting were also extracted from the literature depending on the stage of disease [31].

#### 4.3.4. Transition Probabilities

Incidence rates were extracted from cancer statistics based on the Centers for Disease Control and Prevention [32]. Tumor-unrelated, age-specific death rates of women of all ethnicities in the United States were collected from U.S. Life Tables 2017 [33]. Tumor-related death rates were estimated based on the PREDICT prognostication model [34]. The probability of nodal disease and R0 resection rates were extracted from the literature [35,36].

### 4.4. Economic Analysis

#### 4.4.1. Cost-Effectiveness Analysis

The model-based analyses were conducted using a dedicated decision analysis software (TreeAge Pro 2020, TreeAge Software, Williamstown, MA, USA). A United States healthcare system perspective was selected for this economic evaluation with all costs calculated in US-$. Outcomes were expressed by QALYs. Costs and outcomes were discounted at an annual discount rate of 3.0% in line with current recommendations [26]. A WTP-threshold of $100,000 per QALY was selected [37,38,39]. Using a cycle length of one-year, long-term costs and outcomes were simulated across a time frame of 30 years. All analyses complied with recommendations on the conduct and reporting of cost-effectiveness analyses (Table S3).

#### 4.4.2. Sensitivity Analysis

A deterministic sensitivity analysis was performed to study the impact of changes to single input parameters on the incremental cost-effectiveness ratio (ICER). Costs and diagnostic performance were varied within a plausible range to emphasize their impact; the resulting ICERs were computed and visualized in a tornado diagram (Figure 1).

A probabilistic sensitivity analysis was performed to simulate the overall uncertainty of the input parameters and their combined impact on cost-effectiveness, based on the probability distributions reported in Table 2. A Monte Carlo simulation with 30,000 iterations was applied to investigate the stability of the model outputs.

## 5. Conclusions

Within the limits of the willingness to pay usually applied to a U.S. healthcare system perspective, i.e., $100,000 per QALY gained, AB-MRI can be considered cost-effective in comparison to DBT in screening women at intermediate risk of breast cancer due to high breast density, although various willingness to pay-thresholds have been proposed for the United States and different Western countries.

## Figures and Tables

**Table 1:** Results of base case cost-effectiveness analysis. Discounted average cumulative costs and effects were modeled for a time horizon of 30 years. ICER, incremental cost-effectiveness ratio; QALY, quality-adjusted life-year.

Strategy	Cumulative Costs (US-$)	Incremental Costs (US-$)	Cumulative Effects (QALYs)	Incremental Effects (QALYs)	ICER (US-$/QALY)
**DBT**	8798.47	n/a	19.23	n/a	n/a
**AB-MRI**	9504.77	706.30	19.27	0.03	20,807.04

**Table 2:** Model input parameters.

Variable	Estimation	Source	Distribution
Pre-test probability of malignant lesion	1.59%	Comstock et al. 2020	**β**
Average age at screening	54.9	Comstock et al. 2020	**normal**
Screening interval	two years	Assumption	
Incidence of breast cancer in women with dense breasts	0.40%	Richardson et al. 2016	**β**
Assumed WTP	$100,000	Sanders et al. 2016	
Discount rate	3.00%	Sanders et al. 2016	
**Diagnostic test performances**			
DBT sensitivity	39.1%	Comstock et al. 2020	**β**
DBT specificity	97.4%	Comstock et al. 2020	**β**
AB-MRI sensitivity	95.7%	Comstock et al. 2020	**β**
AB-MRI specificity	86.7%	Comstock et al. 2020	**β**
Full protocol MR mammography specificity	94.0%	Benndorf et al. 2010	**β**
**Costs (Short Term)**			
DBT costs	$214.20	Fleming et al. 2018/ Hunter et al. 2017	**γ**
MRM costs	$314.00	Medicare (CPT code 77047)	**γ**
No further action (true negative)	$0.00	Assumption	
Biopsy	$1536.00	Medicare (CPT code 19083)	**γ**
**Costs (Long Term)**			
Yearly costs without tumor	$0.00	Assumption	
Cost of treatment for tumor < 1 cm	$60,637	Blumen et al. 2015	**γ**
Cost of treatment for tumor > 1 cm	$82,121	Blumen et al. 2015	**γ**
Cost of treatment for advanced stage breast malignancy	$129,387	Blumen et al. 2015	**γ**
**Utilities**			
QOL of patients without detected tumor	1.00	Assumption	
QOL of patients with detected tumor < 1 cm	0.87	Brady et al. 1997	**β**
QOL of patients with detected tumor > 1 cm	0.74	Ahern et al. 2014	**β**
QOL of patients with detected regional breast cancer in an advanced stage	0.62	Polsky et al. 2003	**β**
QOL of patients post simple treatment	0.99	Assumption	**β**
QOL of patients post intensive treatment	0.95	Assumption	**β**
Death	0.00	Assumption	
**Transition probabilities**			
Risk of death without tumor (yearly)	age-adjusted	US Life Tables 2017, women of all ethnicities, Arias et al. 2019	**β**
Risk of death with undetected tumor	10.00% in 10 years	Assumption	**β**
Risk of death with detected < 1 cm tumor	0.11%	NHS Predict	**β**
Risk of death with detected > 1 cm tumor	0.78%	NHS Predict	**β**
Risk of death with detected tumor in advanced stage	1.81%	NHS Predict	**β**
Probability of initial R0 resection < 1 cm	100.00%	Assumption	
Probability of initial R0 resection ≥ 1 cm	90.00%	Lombardi et al. 2019	**β**
Proportion of N+ in < 1 cm tumors	0.00%	Assumption	
Proportion of N+ in > 1 cm tumors	40.00%	Heil et al. 2013	**β**
Proportion of successfully treated tumors < 1 cm if detected within 1 screening interval	100.00%	Assumption	

**Table 3:** Discounted cumulative costs of screening with DBT and AB-MRI (US-$) and the resulting cost-effectiveness expressed by the incremental cost-effectiveness ratio (ICER in US-$ per QALY gained) for varying costs of the diagnostic procedures. AB-MRI, abbreviated breast MRI; DBT, digital breast tomosynthesis.

AB-MRI (US-$)	DBT (US-$)				
	180	200	220	240	260
**210**	8468.90, 8501.48, **959.55**	8661.63, 8501.48, cost-saving	8854.36, 8501.48, cost-saving	9047.09, 8501.48, cost-saving	9239.82, 8501.48, cost-saving
**230**	8468.90, 8694.42, **6643.46**	8661.63, 8694.42, **965.78**	8854.36, 8694.42, cost-saving	9047.09, 8694.42, cost-saving	9239.82, 8694.42, cost-saving
**250**	8468.90, 8887.36, **12,327.37**	8661.63, 8887.36, **6649.69**	8854.36, 8887.36, **972.01**	9047.09, 8887.36, cost-saving	9239.82, 8887.36, cost-saving
**270**	8468.90, 9080.30, **18,011.27**	8661.63, 9080.30, **12,333.59**	8854.36, 9080.30, **6655.92**	9047.09, 9080.30, **978.24**	9239.82, 9080.30, cost-saving
**290**	8468.90, 9273.24, **23,695.18**	8661.63, 9273.24, **18,017.50**	8854.36, 9273.24, **12,339.82**	9047.09, 9273.24, **6662.15**	9239.82, 9273.24, **984.47**
**310**	8468.90, 9466.18, **29,379.08**	8661.63, 9466.18, **23,701.41**	8854.36, 9466.18, **18,023.73**	9047.09, 9466.18, **12,346.05**	9239.82, 9466.18, **6668.38**
**330**	8468.90, 9659.12, **35,062.99**	8661.63, 9659.12, **29,385.31**	8854.36, 9659.12, **23,707.64**	9047.09, 9659.12, **18,029.96**	9239.82, 9659.12, **12,352.28**
**350**	8468.90, 9852.06, **40,746.89**	8661.63, 9852.06, **35,069.22**	8854.36, 9852.06, **29,391.54**	9047.09, 9852.06, **23,713.86**	9239.82, 9852.06, **18,036.19**

## Data Availability

The datasets used and/or analyzed during the current study are available from the corresponding author on reasonable request.

## Supplementary Materials

The following are available online at https://www.mdpi.com/2072-6694/13/6/1241/s1, Table S1: Deterministic sensitivity analysis of the incremental cost-effectiveness ratio (ICER) of AB-MRI compared to DBT in the base-case scenario, Table S2: Cost-effectiveness acceptability analysis, Table S3: Consolidated Health Economic Evaluation Reporting Standards (CHEERS)-checklist.

## Abbreviations

AB-MRI	Abbreviated breast MRI
DBT	Digital breast tomosynthesis
ICER	Incremental cost-effectiveness ratio
MRM	MR-mammography
MRI	Magnetic resonance imaging
PSA	Probabilistic sensitivity analysis
QALY	Quality-adjusted life-year
QOL	Quality of life
WTP	Willingness to pay
